# Kinetics of T lymphocyte subsets and B lymphocytes in response to immunostimulants in flounder (*Paralichthys olivaceus*): implications for CD4^+^ T lymphocyte differentiation

**DOI:** 10.1038/s41598-020-69542-6

**Published:** 2020-08-14

**Authors:** Jing Xing, Hong-fei Tian, Xiao-qian Tang, Xiu-zhen Sheng, Wen-bin Zhan

**Affiliations:** 1 Laboratory of Pathology and Immunology of Aquatic Animals, KLMME, Ocean University of China, Qingdao, 266003 People’s Republic of China; 2 Laboratory for Marine Fisheries Science and Food Production Processes, Qingdao National Laboratory for Marine Science and Technology, No. 1 Wenhai Road, Aoshanwei Town, Qingdao, People’s Republic of China

**Keywords:** Cell biology, Immunology

## Abstract

CD4^+^ T lymphocytes play crucial roles in the adaptive immune system. CD4, as the most effective marker to delineate the T-helper subsets, was identified in many fish species. Two CD4 homologs, CD4-1 and CD4-2, have been reported in flounder (*Paralichthys olivaceus*). In this study, monoclonal antibodies (mAbs) against CD4-1 and CD4-2 of flounder were produced, CD4^+^ T lymphocytes were isolated and identified, and the variations in CD4^+^ and CD8^+^ T lymphocytes and IgM^+^ B lymphocytes after Poly I:C, PMA or β-glucan stimulation were investigated. Then, the expression of transcription factors and cytokines in sorted CD4^+^ T lymphocytes was analyzed. The results showed that the mAbs were specific to flounder CD4-1^+^ and CD4-2^+^ T cells. CD4-1^+^ and CD4-2^+^ cells responded to all three stimulants, while CD8^+^ T lymphocytes only give a strong response to Poly I:C, and the percentages of IgM^+^ B lymphocytes showed a tendency to increase. After stimulation, the expression of transcription factors and cytokines of Th1, Th2 and Th17 cells varied in CD4^+^ T cells. These results will provide crucial foundations for the differentiation and function of teleost CD4^+^ T lymphocytes.

## Introduction

CD4^+^ T cells, also known as T helper (Th) cells, play essential roles in the function of the immune system^1,2^. They display extensive functional and phenotypic diversity in response to pathogens and promote cancer surveillance and tolerance to “self” antigens and environmental allergens^2^. These functions are performed through specific antigen (Ag) recognition by CD4^+^ T cells and subsequent secretion of effector and regulatory cytokines^3^. Upon Ag encounter, the T cell co-receptor CD4 present on T helper cells binds to the peptide/MHC (pMHC) class II molecules on antigen-presenting cells and transduces activation signals^4,5^. Activated CD4^+^ Th cells can further differentiate into a variety of effector Th cell subsets, such as Th1, Th2, Th17 cells and regulatory T (Treg) cells^6^, and Th1 and Th2 cells were discovered to be the dominant populations of Th cells^7^.

The differentiation of Th cells towards a Th1 profile occurs in the presence of IL-12 and IFN-γ and is controlled by the master transcription factor T-bet^6^. These Th1 cells secrete effector cytokines such as IFN-γ, TNF-a and IL-2 that stimulate macrophages and cytotoxic T lymphocytes (CTLs), which play important roles in cellular immunity against intracellular pathogens^6^. Th2 cells, which are promoted by IL-4 and induction of GATA-3, produce cytokines IL-4, IL-5 and IL-13 that stimulate B cells to secrete different antibody isotypes and thus control extracellular infections^6,8,9^. Th17 cells produce cytokines, including IL-17, IL-22, and IL-26, and they express the master transcription factor RORγt. They have a role in host defense against extracellular bacteria, fungi, inflammation and autoimmune diseases^10^. Treg cells, which are regulated through Foxp3, have an essential role in regulating Th1-, Th2-, and Th17-type responses and are responsible for peripheral tolerance^11^. Additionally, some new Th cell subpopulations have been characterized, including Th9, Th22 and T follicular helper (Tfh) cells^9,12^. In mammals, the complexity and plasticity of the Th system has become increasingly clear, and the function of Th cell subsets has also been well characterized.

In contrast to mammals, two CD4 genes have been reported in fish species: the fish CD4-1 molecule is similar to the mammalian CD4 molecule and contains four extracellular Ig-like domains, and the CD4-2 molecule contains two or three Ig-like domains^13^. CD4^+^ cells have also been characterized in fugu (*Takifugu rubripes*), ginbuna crucian carp (*Carassius auratus langsdorfii*), zebrafish (*Danio rerio*), Atlantic salmon (*Salmo salar*), rainbow trout (*Oncorhynchus mykiss*) and flounder (*Paralichthys olivaceus*)^13–18^. In ginbuna crucian carp, CD4-1^+^ cells showed a lymphoid morphology, and the gene expression profile was similar to that of mammalian CD4^+^ T cells. Moreover, carp CD4-1^+^ cells showed the ability to proliferate in mixed leukocyte culture (MLC) and respond to a specific Ag^15^. In zebrafish, CD4-1^+^ lymphocytes increase the expression of cytokines and master transcription factors relevant to Th1/Th2-type responses after boosting with a specific antigen^16^. Two CD4^+^ T cell subsets (CD4-1/CD4-2 double-positive and CD4-2 single-positive T cells) were identified in rainbow trout, and upon bacterial infection, they produced equivalent levels of Th1, Th17, and regulatory T cell cytokines^13^. To date, although CD4^+^ T cells have been identified in several fish species, their functions have only been preliminarily explored. Even though some master transcription factors and cytokines have been cloned, there is no direct evidence showing that fish CD4^+^ Th cells can differentiate into distinct effector phenotypes.

In our previous study, CD4^+^ T lymphocytes were originally identified in flounder (*Paralichthys olivaceus*). Three CD4^+^ T cell subsets, CD4-1 single-positive T cells, CD4-2 single-positive T cells and CD4-1/CD4-2 double-positive T cells, were found in different proportions in flounder peripheral blood, spleen and head kidney, and CD4-1^+^/CD4-2^+^ T cells were the majority of CD4^+^ T cells^18^. In addition, dynamic changes in the percentages of CD4^+^ cells were detected as indicators of fish health and vaccine evaluation^19–25^. In this paper, to further explore the function of CD4^+^ Th cells, monoclonal antibodies against the flounder CD4-1 and CD4-2 molecules were developed. The response of T cell subsets and B lymphocytes to three immunostimulants (Poly I:C, PMA and β-glucan) in flounder and the expression of transcription factors and cytokines in CD4^+^ T cells were investigated. The results will provide crucial foundations for the differentiation and function of teleost CD4^+^ T lymphocytes.

## Results

### Selection and synthesis of epitope peptides

**Figure 1:**
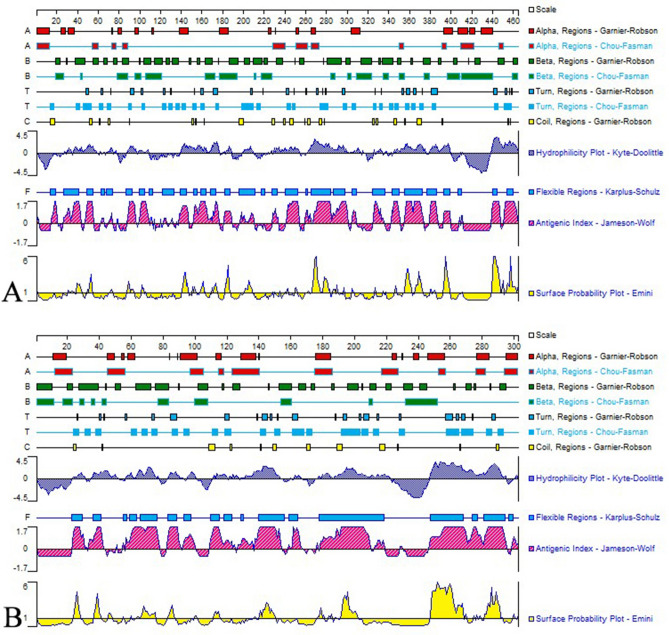
The secondary structures, hydrophilicity, flexibility, antigenicity index and surface probability of flounder CD4-1 (**A**) and CD4-2 (**B**) molecules were analyzed by the DNAStar Protean program.

**Figure 2:**
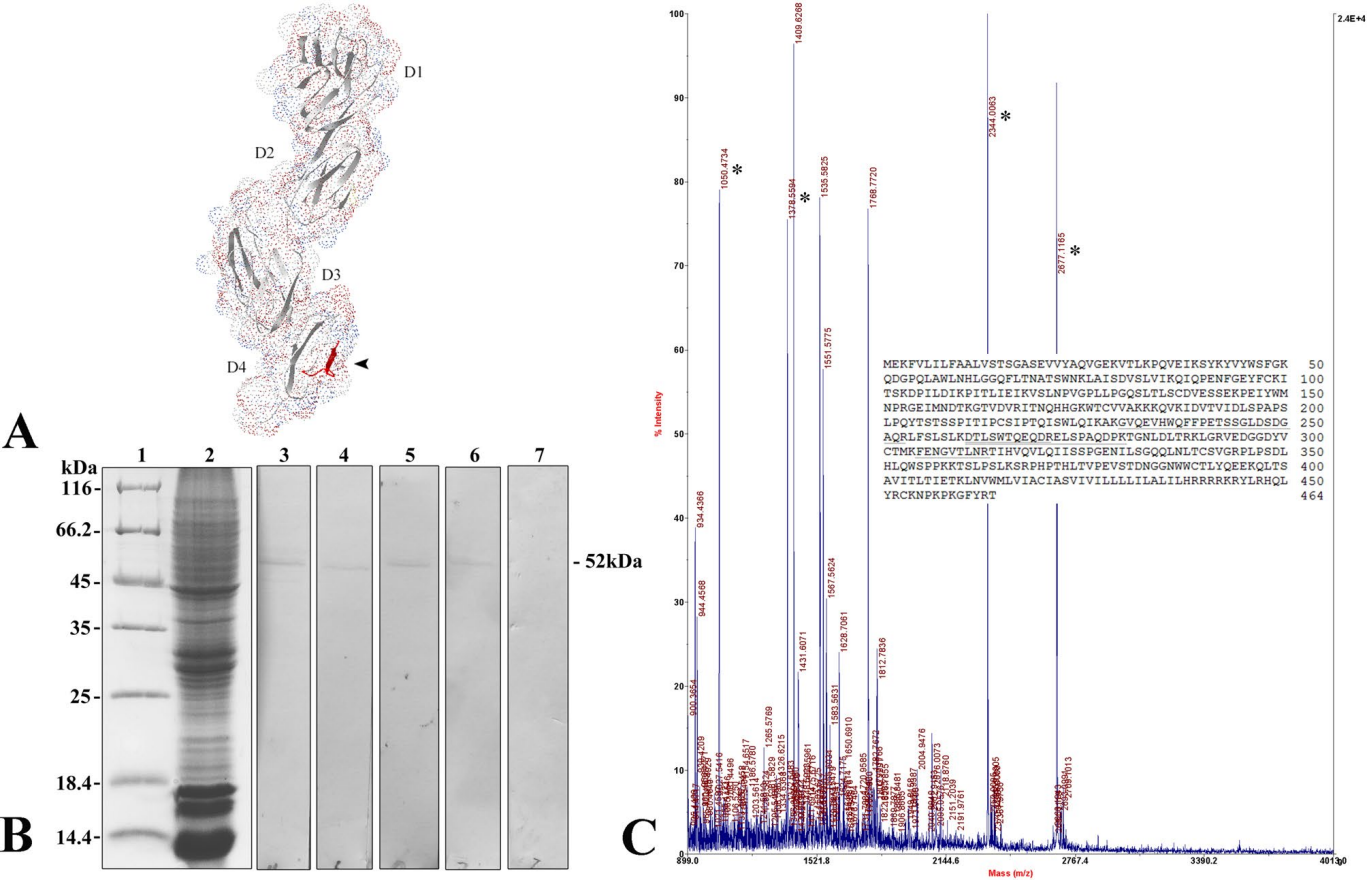
Development of monoclonal antibodies against flounder CD4-1. A. Tertiary structure model of the D1-D4 domain of the flounder CD4-1 molecule predicted by the program Swiss Model Repository Server. The synthetic peptide sequence selected for monoclonal antibody production corresponded to positions 356–369 and is marked in red. B. Western blotting results of the anti-flounder CD4-1 mAb in leukocyte lysates. Lane 1: molecular mass marker; Lane 2: peripheral blood leukocyte lysate; Lane 3: leukocyte lysate incubated with T1-1E12. Lane 4: leukocyte lysate incubated with T1-2A10. Lane 5: leukocyte lysate incubated with T1-2A12. Lane 6: leukocyte lysate incubated with T1-3C12. Lane 7: leukocyte lysate incubated with myeloma culture supernatant as a negative control. C. Mass spectrographic analysis of the immunoreactive band at 52 kDa. LC–MS/MS analysis confirmed that the protein interacting with the mAbs at 52 kDa was the native flounder CD4-1 molecule. The matched four peptides are underlined, and the matched peaks are marked with asterisks.

**Figure 3:**
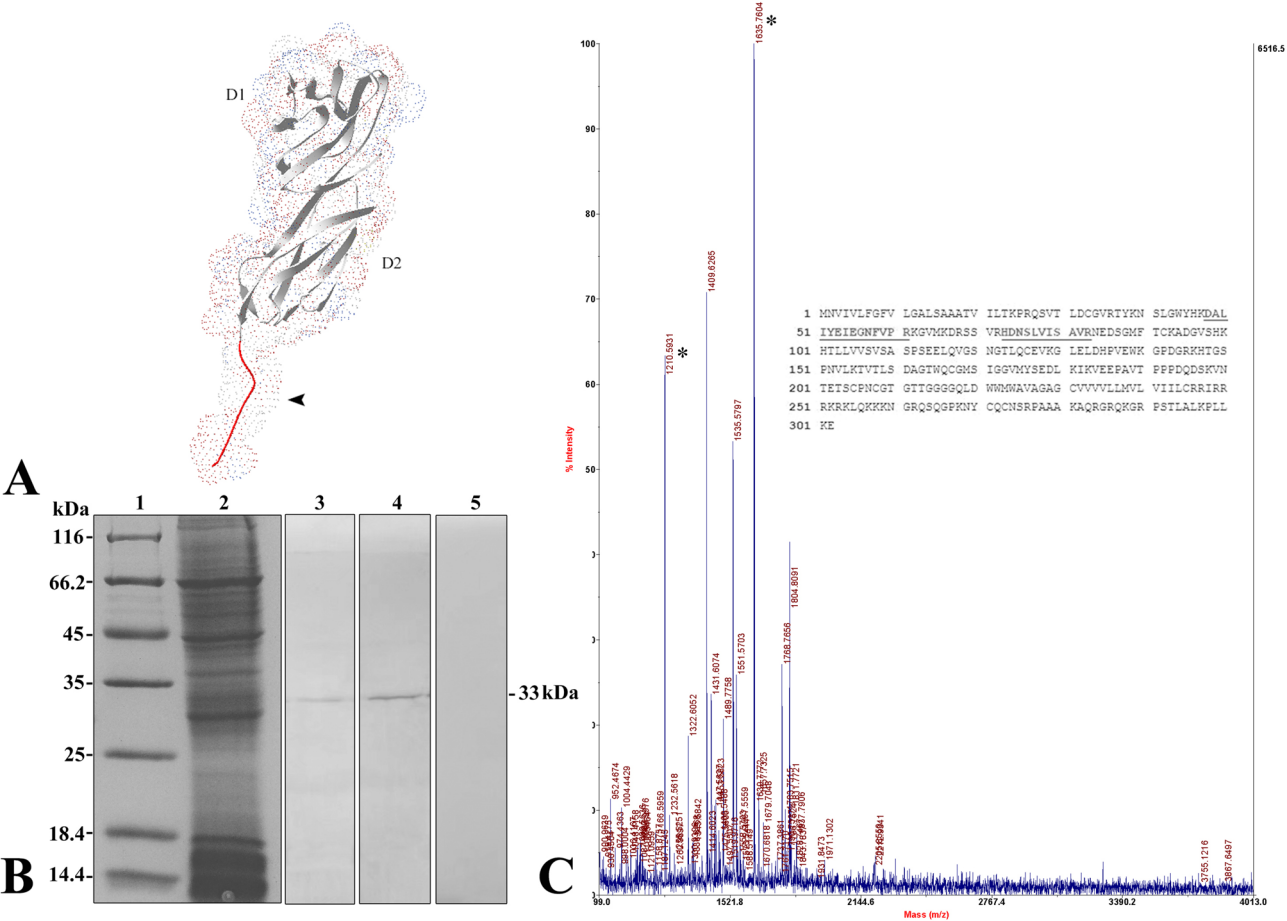
Development of monoclonal antibodies against flounder CD4-2. A. Tertiary structure model of the D1-D2 domain of the flounder CD4-2 molecule predicted by the program Swiss Model Repository Server. The synthetic peptide sequence selected for monoclonal antibody production corresponded to positions 187–200 and is marked in red. B. Western blotting results of the anti-flounder CD4-2 mAb in leukocyte lysates. Lane 1: molecular mass marker; Lane 2: peripheral blood leukocyte lysate; Lane 3: leukocyte lysate incubated with T2-1D8. Lane 2: leukocyte lysate incubated with T2-2A3. Lane 5: leukocyte lysate incubated with myeloma culture supernatant as a negative control. C. Mass spectrographic analysis of the immunoreactive band at 33 kDa. LC–MS/MS analysis confirmed that the protein interacting with the mAbs at 33 kDa was the native flounder CD4-2 molecule. The two matched peptides are underlined, and the matched peaks are marked with asterisks.

Similar to that in mammals, flounder CD4-1 contains a long extracellular region with four Ig-like domains (D1-D4), while flounder CD4-2 contains two Ig-like domains (D1-D2) in the extracellular region. Based on the analysis of amino acid sequences (Fig. 1), a combination of the different parameter results indicated that eleven and five potential immunodominant B-cell epitopes of the flounder CD4-1 and CD4-2 proteins were identified, respectively (Tables 1 and 2). Then, two 14-aa peptides from the potential epitopes were identified with high hydrophilicity, accessibility, flexibility, and antigenicity. The epitope peptide with 14 aa (PPKKTSLPSLKSRP) corresponding to positions 356–369 from the D4 domain of the CD4-1 molecule of flounder was located in the extracellular domain. In addition, the selected peptide sequence was completely exposed on the surface of the flounder CD4-1 structure (Fig. 2A). Similarly, the epitope peptide with 14 aa (PAVTPPPDQDSKVN) corresponding to positions 187–200 from the D2 domain of the CD4-2 molecule of flounder was also located in the extracellular domain (Fig. 3A). Finally, the epitope peptide candidates were synthesized and employed as immunogens to develop mAbs against flounder CD4-1 and CD4-2.

**Table 1:** The potential epitopes of flounder CD4-1; the peptide sequence in bold was selected for monoclonal antibody production.

No	Start	End	Peptide	Length	Average antigenic index
1	14	39	STSGASEVVYAQVGEKVTLKPQVEIK	26	0.28
2	48	56	FGKQDGPQL	9	0.95
3	121	130	LNPVGPLLPG	10	0.50
4	138	174	DVESSEKPEIYWMNPRGEIMNDTKGTVDVRITNQHHG	37	0.56
5	194	219	DLSPAPSLPQYTSTSSPITIPCSIPT	26	0.82
6	239	252	FPETSSGLDSDGAQ	14	0.94
7	263	285	LSWTQEQDRELSPAQDPKTGNLD	23	1.24
8	291	300	GRVEDGGDYV	10	0.69
9	324	332	SSPGENILS	9	0.61
10	351	387	**HLQWSPPKKTSLPSLKSRPHPTHLTVPEVSTDNGGNW**	37	0.95
11	453	463	CKNPKPKGFYR	11	0.89

**Table 2:** The potential epitopes of flounder CD4-2; the peptide sequence in bold was selected for monoclonal antibody production.

No	Start	End	Peptide	Length	Average antigenic index
1	63	73	GVMKDRSSVRH	11	0.54
2	109	125	SASPSEELQVGSNGTLQ	17	0.80
3	136	154	PVEWKGPDGRKHTGSPNVL	19	1.30
4	182	219	**IKVEEPAVTPPPDQDSKVNTETSCPNCGTGTTGGGGQL**	38	1.50
5	254	294	KLQKKKNGRQSQGPKNYCQCNSRPAAAKAQRGRQKGRPSTL	41	1.06

### Production and characterization of mAbs

**Figure 4:**
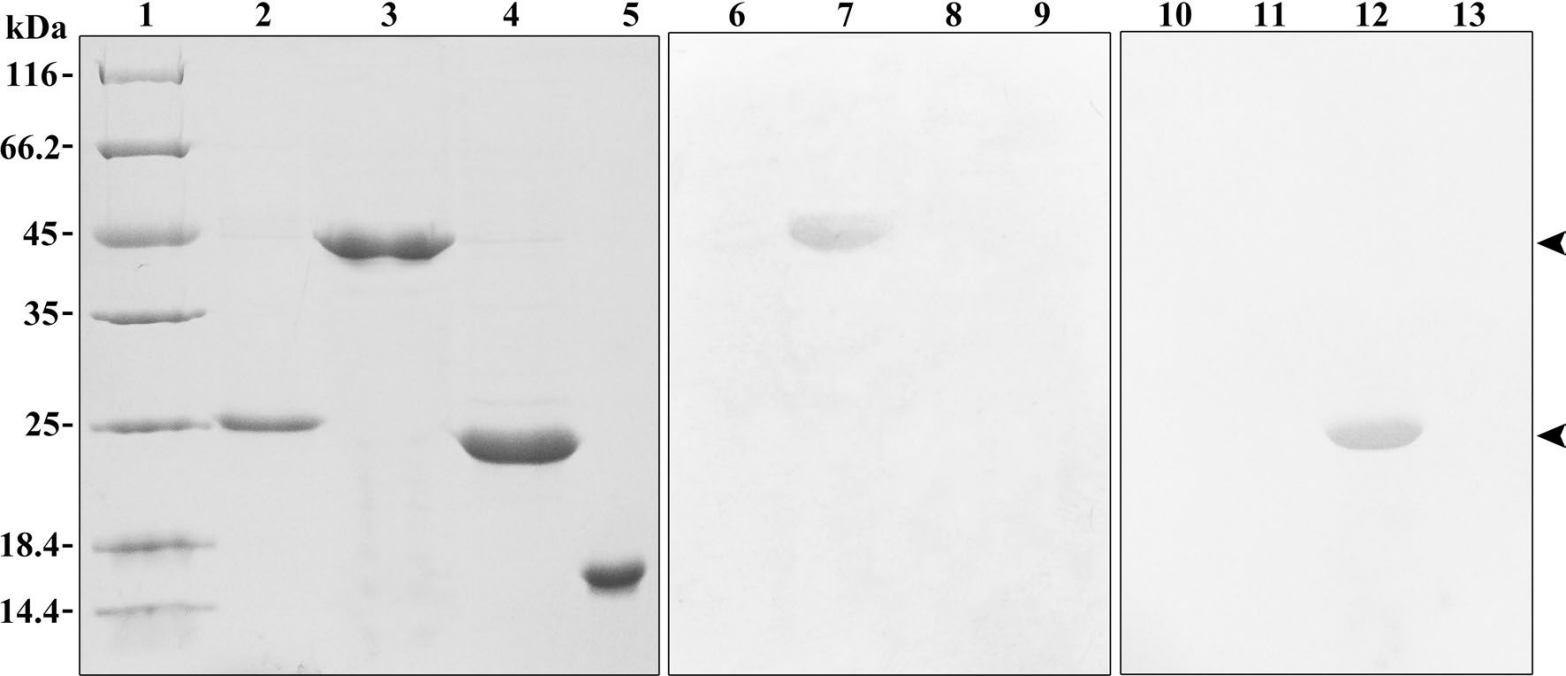
The specificity of monoclonal antibodies analyzed by Western blotting. Lane 1: molecular mass marker; lanes 2,6,10: recombinant protein of flounder CD3ɛ; lanes 3,7,11: recombinant protein of flounder CD4-1; lanes 4,8,12: recombinant protein of flounder CD4-2; lanes 5,9,13: recombinant protein of flounder CD8β; lanes 6–9 were incubated with the mAbs against flounder CD4-1; lanes 10–13 were incubated with the mAbs against flounder CD4-2. The arrow shows the immunoreactive band.

**Figure 5:**
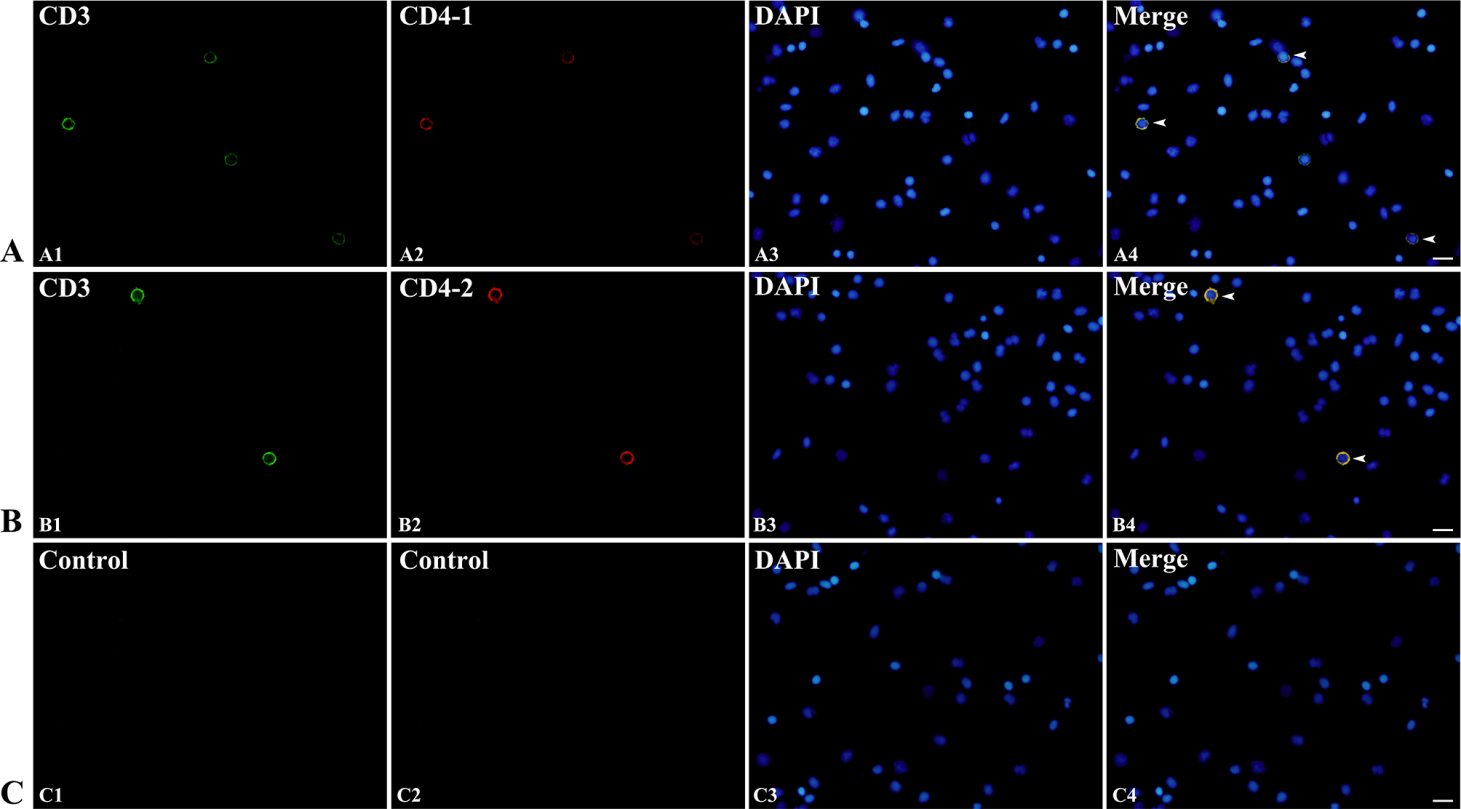
Double immunofluorescence staining results of CD3ɛ^+^/CD4-1^+^ and CD3ɛ^+^/CD4-2^+^ T lymphocytes. A. CD3ɛ^+^/CD4-1^+^ T lymphocytes, A1: CD3ɛ^+^, A2: CD4-1^+^, A3: DAPI, A4: Merge. B. CD3ɛ^+^/CD4-2^+^ T lymphocytes, B1: CD3ɛ^+^, B2: CD4-2^+^, B3: DAPI, B4: Merge. C. Negative controls. Rabbit anti-flounder CD3ɛ Abs and mouse anti-flounder CD4-1 and CD4-2 mAbs were used in this experiment. White arrows indicate double-positive cells. Bar = 10 µm.

**Figure 6:**
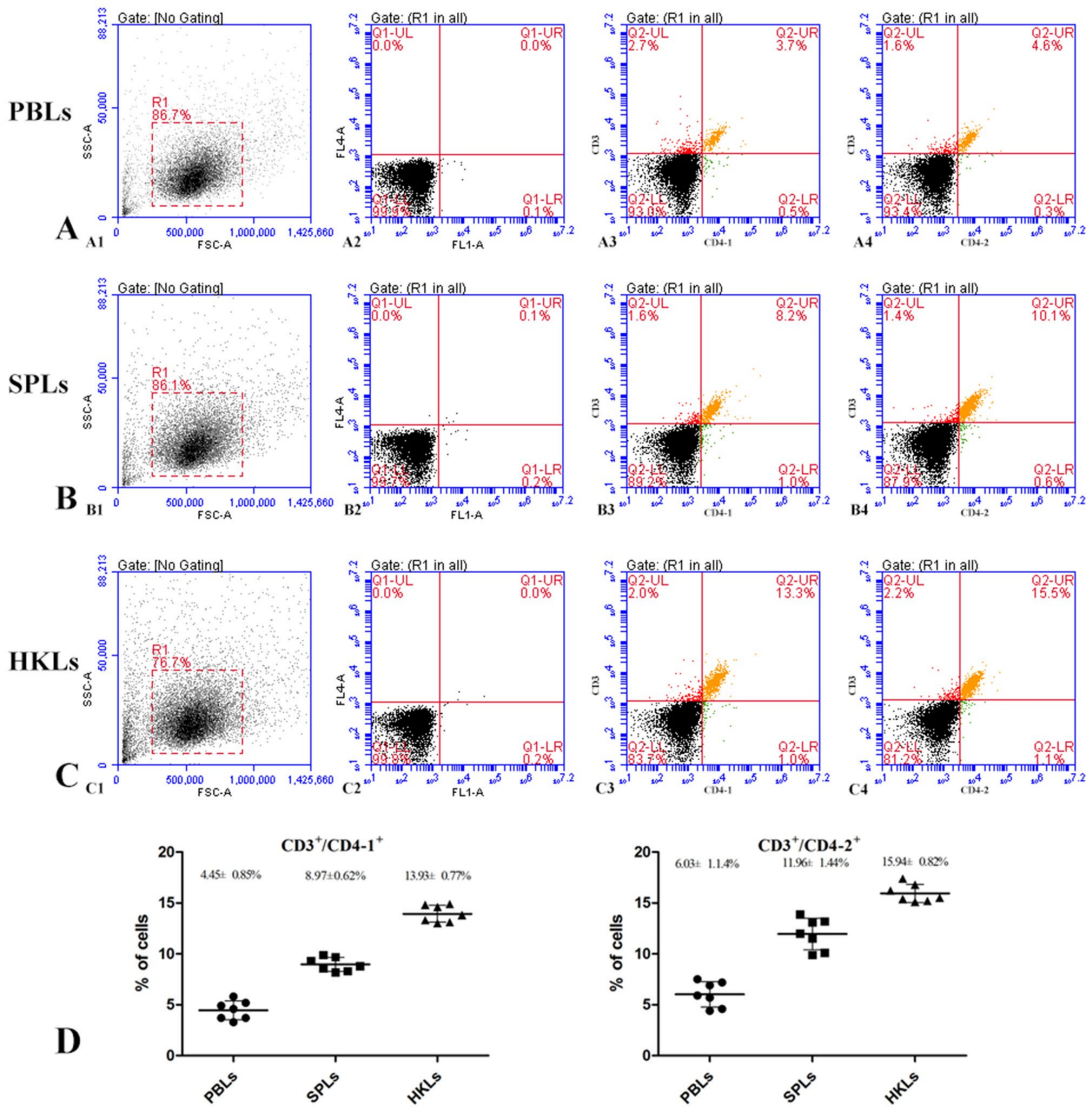
Two-color flow cytometry results of CD3^+^/CD4-1^+^ and CD3^+^/CD4-2^+^ T lymphocytes in peripheral blood leukocytes, spleen, and head kidney. FSC area (FSC-A)/SSC area (SSC-A) analyses are shown in A1, B1, C1, and the red gate represents lymphocyte cells. A2, B2, C2 represent the negative controls. A3, B3, C3: CD3^+^/CD4-1^+^ T lymphocytes in peripheral blood leukocytes, spleen and head kidney, respectively. A4, B4, C4: CD3^+^/CD4-2^+^ T lymphocytes in peripheral blood leukocytes, spleen and head kidney, respectively. D. Percentages of CD3^+^/CD4-1^+^ and CD3^+^/CD4-2^+^ T lymphocytes; data represent the mean ± SD.

After cell fusion, four hybridomas, designated T1-1E12, T1-2A10, T1-2A12 and T1-3C12, which gave strongly positive results to the CD4-1 peptide, were selected. Then, these hybridomas were cloned by limiting dilution. Western blotting results showed that all four mAbs bound to a 52 kDa protein from leukocyte lysates, which was in accordance with the molecular mass of the flounder CD4-1 molecule (Fig. 2B). In contrast, no band was found when the mAbs were replaced by myeloma culture supernatant. Subsequently, the mass spectrometry results showed that the 52 kDa protein was identified as a native flounder CD4-1 molecule, in which 4 peptides were matched to the chain of flounder CD4-1 with 11% coverage of the amino acid sequence (Fig. 2C). In addition, two mAbs against the flounder CD4-2 molecule, designated T2-1D8 and T2-2A3, were successfully developed. The mAbs could react to a 33 kDa protein from leukocyte lysates (Fig. 3B), which was proven to be a native flounder CD4-2 molecule (Fig. 3C). Two peptides were matched in the chain of flounder CD4-2 with 8% coverage of the amino acid sequence. The results of cross reactivity experiments showed that the mAbs against CD4-1 could specifically recognize the recombinant protein of flounder CD4-1 at a molecular weight of 45 kDa. The mAbs against CD4-2 could specifically recognize the recombinant protein of flounder CD4-2 at a molecular weight of 25 kDa. No cross-reaction with the recombinant protein of flounder CD3, CD4-2 or CD8β was detected (Fig. 4). In addition, CD3ɛ^+^/CD4-1^+^ and CD3ɛ^+^/CD4-2^+^ T lymphocytes were observed under a fluorescence microscope (Fig. 5). The proportion of CD3ɛ^+^/CD4-1^+^ and CD3ɛ^+^/CD4-2^+^ T lymphocytes varied in the peripheral blood, spleen and head kidney, and very few CD3ɛ^-^/CD4-1^+^ and CD3ɛ^-^/CD4-2^+^ cells were detected (Fig. 6).

### Isolation of CD4^+^ T lymphocytes

**Figure 7:**
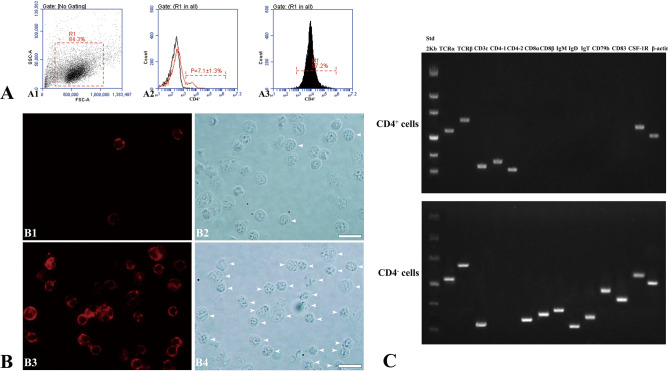
Isolation of CD4^+^ T lymphocytes in flounder. A. Flow cytometric analysis of the purity of sorted CD4^+^ cells. A1. Side-scatter (SSC)/forward-scatter (FSC) dot plots in peripheral blood leukocytes. A2. Flow cytometry detection of CD4^+^ cells in peripheral blood leukocytes. The peripheral blood leukocytes were incubated with a mixture of mAbs against CD4-1 and mAbs against CD4-2. A3. Flow cytometry detection of the purity of CD4^+^ cells after immunomagnetic bead sorting. B. Immunofluorescence staining analysis of the purity of sorted CD4^+^ cells. B1 and B2. Immunofluorescence staining and differential interference contrast results of unsorted CD4^+^ cells. B3 and B4. Immunofluorescence staining and differential interference contrast results of sorted CD4^+^ cells. The white arrows indicate positive CD4^+^ cells. Bar = 10 µm. C. RT-PCR analysis of the expression of marker genes in CD4^+^ cells and CD4^-^ cells.

Most of lymphocytes of flounder were gated based on the size and granularity on the forward scatter (FSC)/side scatter (SSC) plots in flow cytometry analysis (Fig. 7A1). The flow cytometry results showed that CD4^+^ cells were 7.1 ± 1.3% of the peripheral blood leukocytes of flounder (Fig. 7A2). After immunomagnetic bead sorting, the purity of CD4^+^ cells reached 96.0 ± 1.2% (Fig. 7A3). Immunofluorescence staining results showed a few red fluorescent signals on the membranes of lymphocytes before cell sorting (Fig. 7B1), and cells were observed by interferential equipment in the same field (Fig. 7B2). After cell sorting, the presence of red fluorescent signals was observed on the membranes of all sorted cells (Fig. 7B3) and cells were also observed by interferential equipment in the same field (Fig. 7B4). These results showed that high-purity flounder CD4^+^ cells were obtained. Moreover, the expression profiles of lymphocyte surface markers in sorted CD4^+^ cells were analyzed (Fig. 7C). The transcripts of TCRα, TCRβ, CD3, CD4-1 and CD4-2 were detected in CD4^+^ cells, whereas the transcripts of CD8, CD83 and surface markers of B cells, such as IgM, IgD, IgT and CD79β, were not found. In contrast, CD8, CD83 and B cell surface markers were found in CD4^-^ cells. Expression of TCR and CD3 was also detected in CD4^-^ cells. Notably, CSF-1R, a cell surface marker of macrophages, was also found to be expressed in CD4^+^ cells.

### Variations in T lymphocyte subsets and B lymphocytes after stimulation

**Figure 8:**
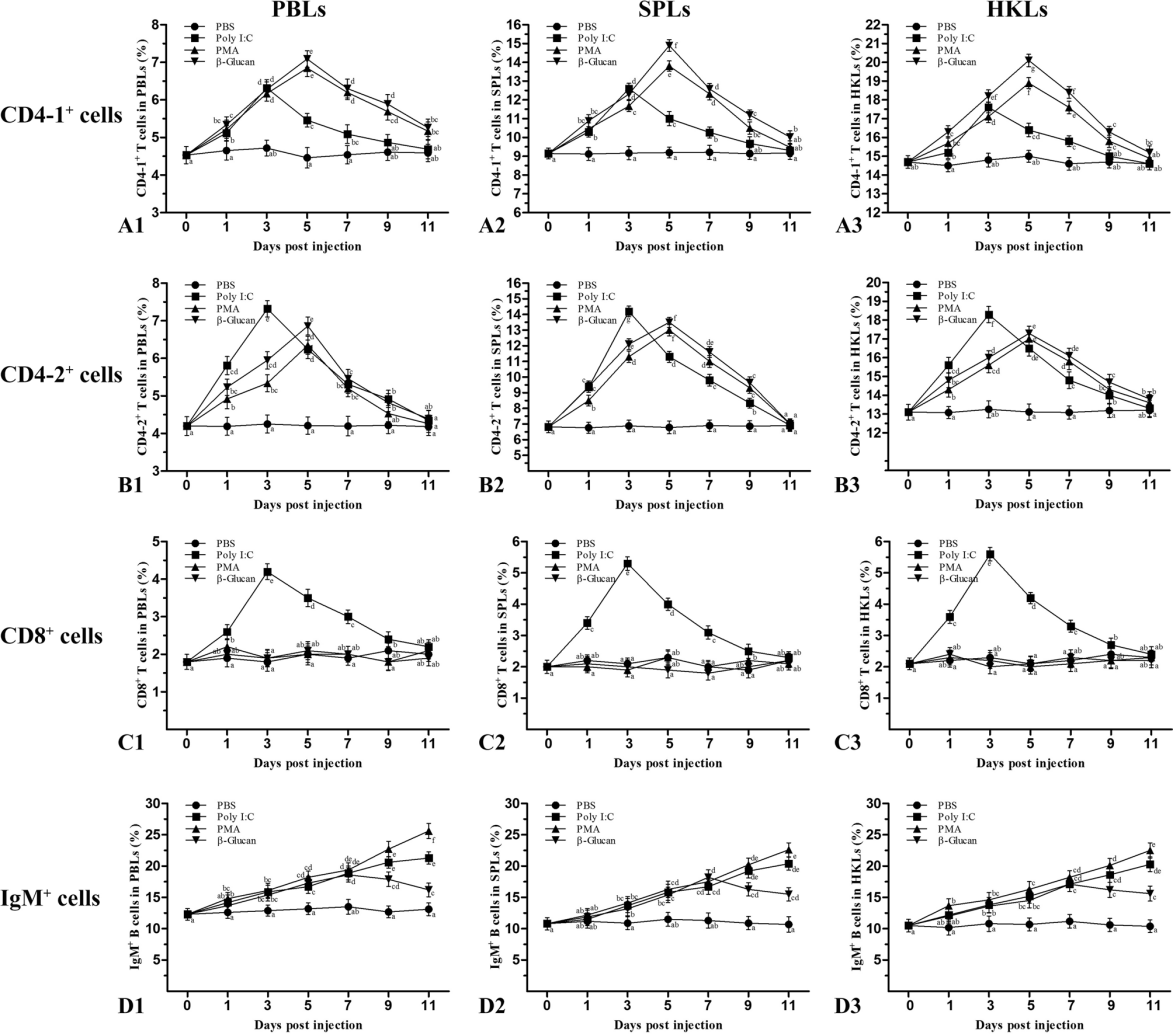
Variations in CD4-1^+^, CD4-2^+^, CD8^+^ T cells and IgM^+^ B cells after stimulation with Poly I:C, PMA or β-glucan. The results are presented as the mean ± SEM (n = 3). Different letters on the bars indicate statistical significance at each time point (*p* < 0.05). A1, A2, and A3: CD4-1^+^ T cells in peripheral blood, spleen and head kidney leukocytes. B1, B2, and B3: CD4-2^+^ T cells in peripheral blood, spleen and kidney leukocytes. C1, C2, and C3: CD8^+^ T cells in peripheral blood, spleen and kidney leukocytes. D1, D2, and D3: IgM^+^ B cells in peripheral blood, spleen and kidney leukocytes.

**Figure 9:**
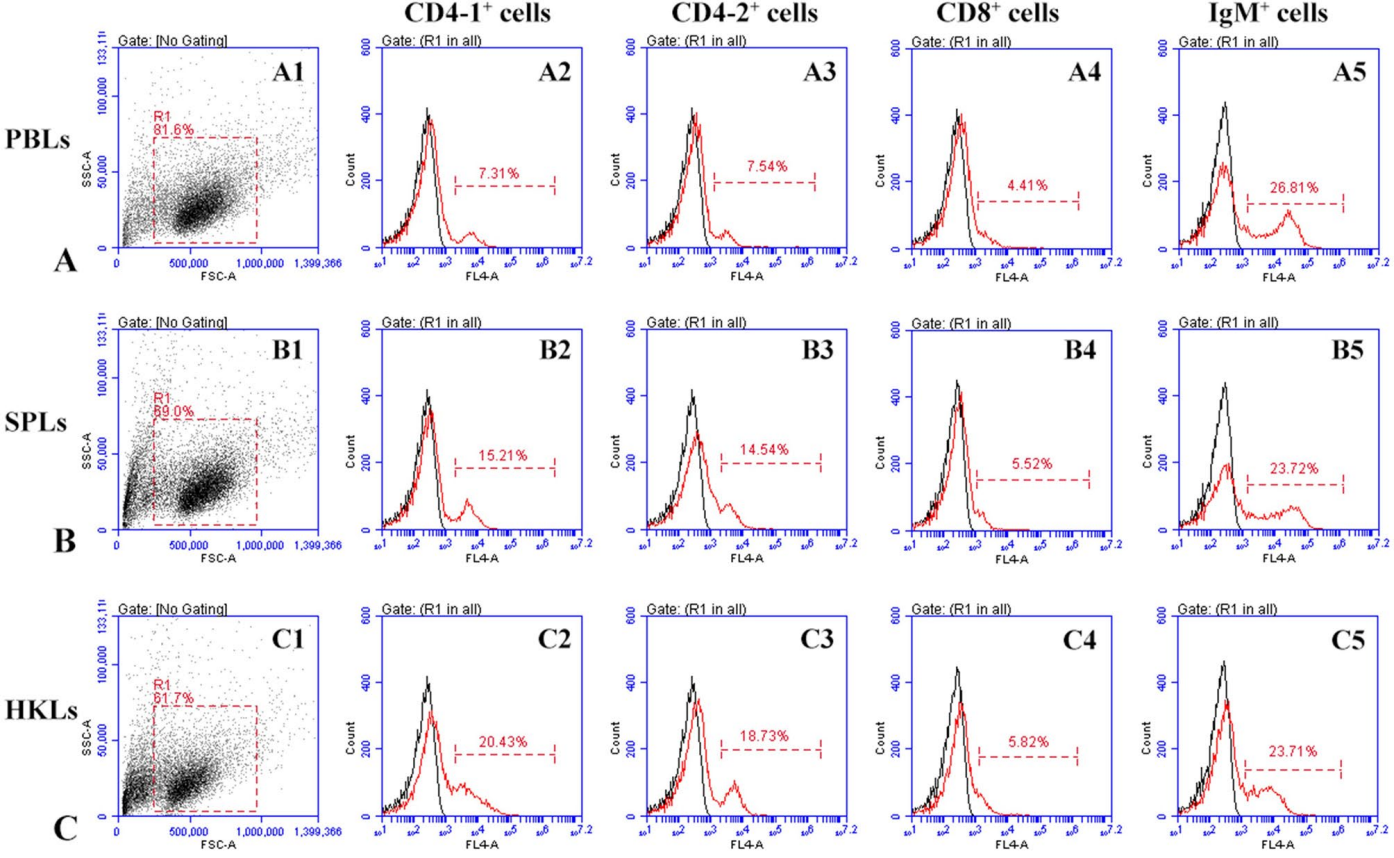
The fluorescence histograms of maximum number of CD4-1^+^, CD4-2^+^, CD8^+^ T cells and IgM^+^ B cells after stimulation with Poly I:C, PMA or β-glucan. A1, B1, and C1: Side-scatter (SSC)/forward-scatter (FSC) dot plots in peripheral blood leukocytes (PBLs), spleen leukocytes (SPLs) and head kidney leukocytes (HKLs), respectively. A2, B2, and C2: The fluorescence histograms of the maximum number of CD4-1^+^ T cells in PBLs, SPLs and HKLs. A3, B3, and C3: The fluorescence histograms of the maximum number of CD4-2^+^ T cells in PBLs, SPLs and HKLs. A4, B4, and C4: The fluorescence histograms of the maximum number of CD8^+^ T cells in PBLs, SPLs and HKLs. A5, B5, and C5: The fluorescence histograms of the maximum number of IgM^+^ B cells in PBLs, SPLs and HKLs.

After Poly I:C, PMA and β-glucan injection, the variations in CD4-1^+^, CD4-2^+^, and CD8β^+^ T cells and IgM^+^ B cells in peripheral blood, spleen and head kidney were analyzed and are shown in Fig. 8. The percentages of CD4-1^+^ and CD4-2^+^ cells in the three tissues showed a tendency to initially increase followed by a decrease to near-normal levels. The proliferation of CD4-1^+^ and CD4-2^+^ cells appeared on the 1st day, peaked on the 3rd or 5th day (*p* < 0.05), and then declined on the 7th day in the three groups. Remarkably, in the Poly I:C group, the percentages of CD4-1^+^ and CD4-2^+^ cells reached the peak level earlier, and the percentages of CD4-2^+^ cells in the peak were higher than those in the other two groups. CD8^+^ T lymphocytes showed a strong response to Poly I:C on the 1st day and reached a peak on the 3rd day (*p* < 0.05). In the PMA and β-glucan groups, no significant increase in CD8^+^ T cells was detected in peripheral blood, spleen or head kidney. The percentages of IgM^+^ B lymphocytes in the Poly I:C and PMA groups showed a tendency to increase gradually until the end of the sampling period. In the β-glucan group, the percentages of IgM^+^ B cells increased on the 1st day, peaked on the 7th day (*p* < 0.05), and then gradually decreased in the three tissues. In the control group, the percentages of CD4-1^+^, CD4-2^+^, and CD8β^+^ T cells and IgM^+^ B cells maintained a consistent level during the experimental period. Additionally, the gated lymphocytes in SSC/FSC dot plots and fluorescence histograms for the maximum number of CD4-1^+^, CD4-2^+^, and CD8β^+^ T cells and IgM^+^ B cells in peripheral blood, spleen and head kidney are shown in Fig. 9.

### Expression of transcription factors and cytokines in CD4^+^ cells after stimulation

**Figure 10:**
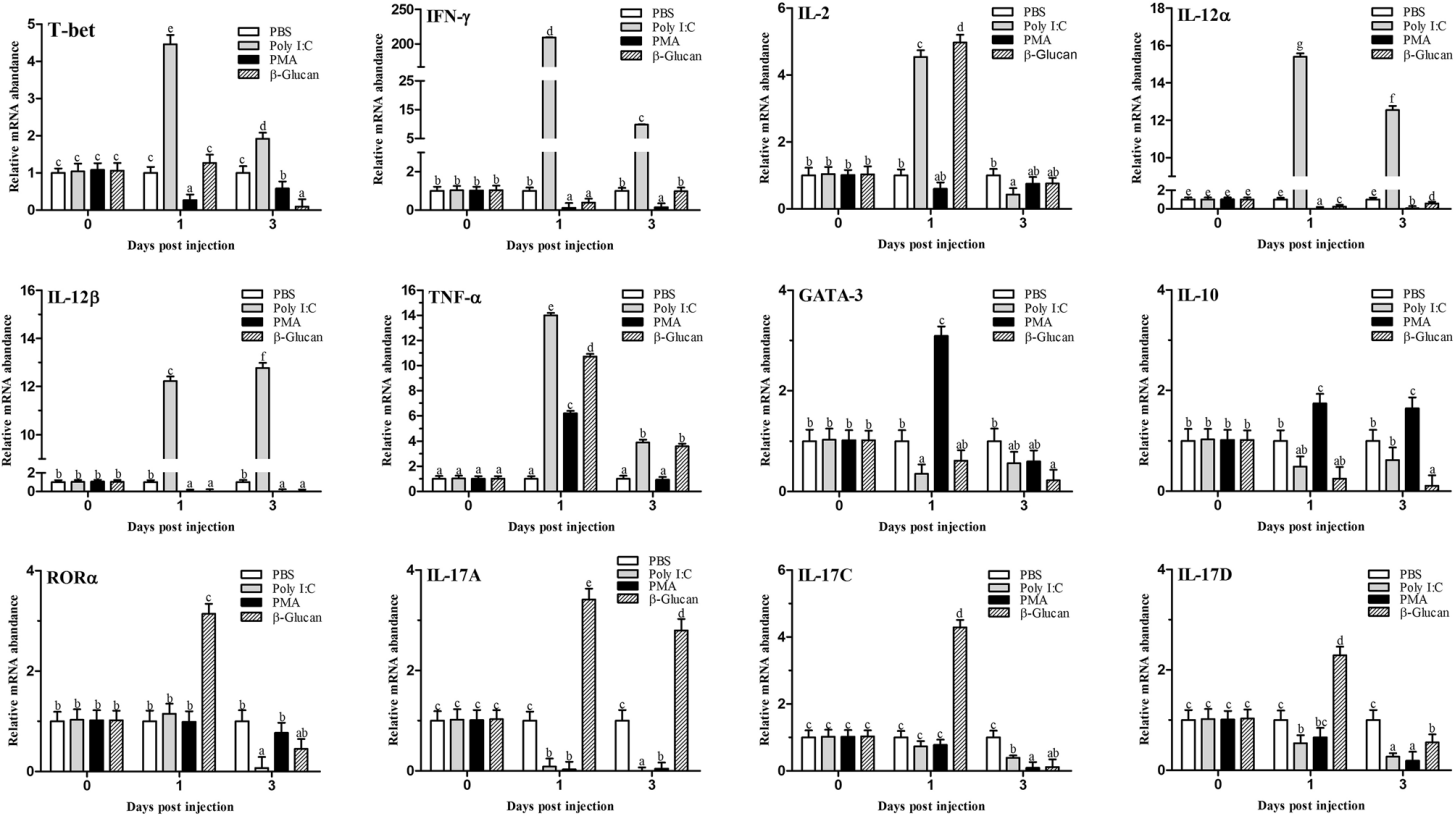
Analysis of the expression of transcription factors and cytokines in CD4^+^ cells after stimulation with Poly I:C, PMA or β-glucan. The mRNA level of each gene was normalized to that of 18S rRNA. For each gene, the mRNA level of the control fish was set as 1. The results are expressed as the mean ± SEM (n = 3). Different letters on the bar represent statistical significance (*p* < 0.05).

After stimulation with Poly I:C, PMA and β-glucan, some transcription factors of T-helper (Th) cells and several cytokine gene expression profiles in flounder CD4^+^ cells were analyzed, and the results are shown in Fig. 10. T-bet, the master transcription factor of Th1 cells, IFN-γ, IL-2, IL-12 and TNF-α, which are associated with Th1 cytokines in mammals, were significantly increased following stimulation with Poly I:C (*p* < 0.05). The IFN-γ gene was increased more than 200-fold on the 1st day post Poly I:C stimulation (*p* < 0.05). The transcription factors of Th2 cells and Th17 cells, GATA-3 and RORα, were decreased after Poly I:C stimulation. The expression of IL-10, IL-17A, IL-17C and IL-17D was also decreased in the Poly I:C group. PMA stimulation upregulated the TNF-α, GATA-3 and IL-10 genes compared to the PBS treatment. However, other genes were decreased after stimulation with PMA. In the β-glucan group, IL-2, TNF-α, RORα, IL-17A, IL-17C and IL-17D were strongly increased compared to those in the control group, while other genes were suppressed.

## Discussion

CD4^+^ cells play critical roles in the adaptive immune system. However, very few functional studies have been carried out on fish CD4^+^ cells due to a lack of tools. Antibodies for cell surface markers are one of the most important tools in leukocyte identification and function^17^. Recently, monoclonal antibodies against CD4 molecules were generated for ginbuna crucian carp and rainbow trout^13,15^. CD4^+^ cells have been identified using monoclonal antibodies, and the function of teleost CD4^+^ cells was demonstrated to be similar to that of mammalian Th cells. However, more investigations on the characterization of CD4^+^ cells in various fish species are still needed because of the unique features of the fish immune system. Hence, in this study, monoclonal antibodies (mAbs) against flounder (*P. olivaceus*) CD4-1 and CD4-2 were successfully developed, and CD4^+^ T lymphocytes were isolated and identified from peripheral blood.

Peptide antibodies are powerful tools and are widely used in biological experiments^26^. Recently, some studies reported that peptide antibodies were also used to label fish leukocyte surface molecules. Boardman et al. produced a mAb against the rainbow trout CD3ε chain by a synthetic peptide and used it to elucidate both the distribution and role of T lymphocytes in the immune system^27^. Two peptide sequences with high hydrophilicity were chosen for the production of polyclonal antiserum and successfully used for monitoring zebrafish CD4-1^+^ lymphocyte responses^16^. Moreover, it was reported that polyclonal antibodies against a 10-aa synthetic peptide of the salmon CD4-1 sequence were produced to identify the CD4-1^+^ subpopulation of T cells^17^. These studies encouraged us to develop antibodies against synthetic peptides of some cell-surface molecules in fish, and the antibodies would be good tools in identifying the cells at the molecular level and exploring their functions. In the present study, based on the bioinformatics analysis of amino acid sequences, two 14-aa epitope peptide candidates were identified and employed as immunogens to develop mAbs against flounder CD4-1 and CD4-2. Although polyclonal antibodies against recombinant CD4-1 and CD4-2 proteins of flounder were produced, respectively, the dynamic change in the percentages of CD4^+^ cells was used as an indicator of the fish health status and vaccine evaluation in our lab^19–25^. However, some characteristics of polyclonal antibodies make them incomparable to monoclonal antibodies, and mAbs against CD4 molecules are sustainable and powerful tools in the functional study of fish CD4^+^ T cells. Finally, four CD4-1 mAbs were produced that responded to lymphocyte lysates at 52 kDa. Moreover, the 52 kDa protein in leukocytes was recognized by the mAbs, and it was identified as the native flounder CD4-1 molecule. Similarly, two CD4-2 mAbs reacted to a band at 33 kDa from lymphocyte lysates, which was proven to be the native flounder CD4-2 molecule. The mAbs also specifically bound to recombinant flounder CD4-1 and CD4-2 proteins. CD3, a surface marker of T lymphocytes, was expressed on CD4-1^+^ and CD4-2^+^ cells in flounder. Very few CD3ɛ^-^/CD4-1^+^ and CD3ɛ^-^/CD4-2^+^ cells were observed, which was indicative of CD4 expressed on T lymphocytes in flounder. However, the proportions of CD3^+^/CD4-1^+^ and CD3^+^/CD4-2^+^ T cells were relatively lower than the results we obtained previously^18^. We consider the reason to be related to the stronger specificity of monoclonal antibodies. These results indicate that the mAbs developed in this study are specific in identifying flounder CD4-1^+^ and CD4-2^+^ T cells.

In our previous study, three CD4^+^ lymphocyte populations (CD4-1^+^/CD4-2^-^ (CD4-1SP), CD4-1^-^/CD4-2^+^ (CD4-2SP) and CD4-1^+^/CD4-2^+^ (CD4DP) cells) were detected in flounder with two-color flow cytometry^18^. The separation of the three CD4^+^ lymphocyte populations will be more conducive to further analysis of the characteristics of flounder CD4^+^ T lymphocyte subsets. However, due to technical limitations, it was difficult to separate the three CD4^+^ T cell subsets for further research. In the future, we will consider using single-cell sequencing technology to further explore CD4^+^ cells in flounder. Therefore, we focused on total flounder CD4^+^ T lymphocytes in further experiments. To detect all CD4^+^ cells in peripheral blood leukocytes, the leukocytes were incubated with a mixture of the CD4-1 and CD4-2 mAbs as primary antibodies. Approximately 7% of CD4^+^ T cells were detected in flounder peripheral blood leukocytes, and total CD4^+^ cells were isolated using the immunomagnetic bead separation method. A high level of purity (96%) for sorted CD4^+^ cells was confirmed by flow cytometry, immunofluorescence and RT-PCR. The TCR and CD3 genes were expressed both in CD4^+^ and CD4^-^ cells, indicating that some T cells did not express CD4 molecules, which may be CD8^+^ T cells. CD8 gene expression was clearly detected in CD4^-^ cells. Similarly, some cell surface markers of B cells, such as IgM, IgD, IgT and CD79, were not found in CD4^+^ cells but were detected in CD4^-^ cells. CD83, a standard lineage maker for activated or differentiated DCs^28^, was expressed on CD4^-^ cells.

The surface marker of monocytes/macrophages CSF-1R^29^ was detected both in flounder CD4^+^ and CD4^-^ cells. Interestingly, macrophage markers were found in CD4^+^ cells of flounder. In rainbow trout, CD4-1SP cells are mainly myeloid with the highest phagocytic activity and capacity^13^. However, our study showed that the flounder CD4-1 molecule can colocalize with the CD3 molecule, which is generally considered to be a marker of T lymphocytes. In other words, we do not have sufficient evidence for the existence of CD4-1^+^ myeloid cells in flounder. In our previous study, mAbs against flounder IgM were used to characterize the lymphocyte population size and granularity on forward scatter (FSC)/side scatter (SSC) plots in flow cytometry analysis. Giemsa staining also showed that the lymphocyte populations represented a high proportion of the isolated leukocytes in our lab^30^. Hence, a small number of myeloid cells may be present in our isolated leukocytes. To prove the existence of flounder CD4^+^ myeloid cells, we subsequently adjusted the Percoll density and centrifugal force to change the separation method of leukocytes to obtain more myeloid cells and not lymphocytes. In this experiment, it can be speculated that a small number of monocytes/macrophages contaminated the isolated CD4^+^ T lymphocytes or a small number of CD4^+^ myeloid cells was present in the isolated CD4^+^ cells. In the future, we will attempt to explore the existence of CD4^+^ myeloid cells in flounder because CD4 molecules have also been found to be expressed on macrophages in humans and rats^31,32^.

In mammals, after antigen stimulation, CD4^+^ cells can differentiate into different subpopulations, such as Th1, Th2, Th17 cells and regulatory T (Treg) cells^6^. The effector T lymphocyte subsets played different roles in immune responses through subsequent secretion of effector and regulatory cytokines^17^. However, a small number of studies have shown evidence that fish CD4^+^ Th cells can differentiate into distinct effector phenotypes. The differentiation of CD4^+^ Th cells can also be regulated by some stimulants through signaling pathways. Therefore, in this paper, Poly I:C, PMA and β-glucan, three kinds of stimulants with diverse mechanisms of action, were injected into flounder. Then, the variations in CD4^+^ and CD8^+^ T cell subsets and IgM^+^ B cells were investigated, and the expression of transcription factors and cytokines in sorted CD4^+^ T lymphocytes was analyzed. Poly I:C is a synthetic analog of viral double-stranded RNA (dsRNA) that has been shown to induce Th1 responses^33^. PMA is a potent tumor inducer that can facilitate the activation of TNF-α, NF-κB and AP1 (activator protein 1) and has been shown to induce a Th2 lymphocyte cell response^34^. β-glucan can activate many signaling pathways upon binding to β-glucan receptors (Dectin-1, CD5 and complement receptor 3) and has been shown to be capable of inducing Th17 responses^35^. In this study, CD4-1^+^ and CD4-2^+^ T cells proliferated in response to Poly I:C, PMA and β-glucan, suggesting that flounder CD4^+^ T lymphocytes can differentiate into different effector Th cell subsets and participate in Th-related immune responses. Previously, the CD4-2 mRNA level was found to be dramatically upregulated after *E. tarda* and VHSV infection in flounder, indicating that CD4-2^+^ cells are involved in Th1-related immune responses and have a major role against intracellular microorganisms^36^. In the Poly I:C group, the percentages of CD4-2^+^ cells reached the peak level earlier, and the percentages of CD4-2^+^ cells in the peak were higher than those in the other two groups, also indicating that CD4-2^+^ cells are involved in the Th1-related immune response in flounder.

Although the variations in CD4-1^+^ and CD4-2^+^ T cells were analyzed after stimulation by one-color flow cytometry, bioparametric analysis should be performed to investigate the different immune responses of flounder CD4-1SP, CD4-2SP and CD4DP T cells. In this study, mAbs against flounder CD4-1 and CD4-2 were generated and to prove their specificity, the mAbs were only used in indirect immunofluorescence experiments. In the future, we will label the mAbs to meet more experimental requirements. The immune responses of the different identified CD4^+^ T cell subsets to various antigens were investigated, and the dynamic changes in the percentages of CD4^+^ T cells were used as indicators of the health status and vaccine evaluation in flounder. However, further study about the differentiation and effector function of flounder CD4^+^ T cells is needed. In ginbuna crucian carp, CD4-1^+^ T cells showed a lymphoid morphology and had the ability to proliferate in mixed leukocyte culture (MLC) and respond to a specific antigen. These results suggest that carp CD4-1^+^ T cells are equivalent to helper T lymphocytes in mammals^15^. In salmon, CD8α, CD8β and IgM transcripts were also detected in highly purified CD4^+^ cells, but this result was not explained^17^. Interestingly, zebrafish CD4-1 and CD4-2 molecules were expressed not only in lymphocytes but also in precursor cells and monocytes/macrophages^16^. Similarly, CD4-1SP myeloid cells were also identified and characterized in rainbow trout^13^. These results suggest that further studies are needed to investigate the characteristics and functions of CD4^+^ cells in different fish species.

With the deepening of research, teleost CD8^+^ T cells have been functionally identified as cytotoxic T lymphocytes (CTLs), which kill virus-infected cells and transplanted allogeneic cells and tissues^1^. In the present study, CD8^+^ T cells proliferated significantly after stimulation with Poly I:C, while no significant increase in CD8^+^ cells was observed in the other groups. IFN-γ, an effector cytokine of Th1 cells, can activate macrophages and enhance their ability to kill phagocytic pathogens^37^. Moreover, IFN-γ can also collaborate with IL-2 to increase the proliferation and differentiation of CTLs^38^. For this reason, CD8^+^ T cells were analyzed in this study, and the results indicate that CD4^+^ Th cells can recruit CD8^+^ cells involved in cellular immunity against intracellular microorganisms^39^. In mammals, Th2 cells produce IL-4, IL-5, and IL-13, which stimulate B cells to secrete antibodies to control helminths and other extracellular pathogens^6^. In this study, the percentages of IgM^+^ B cells were observed to respond to three stimulants, and the percentages of IgM^+^ B lymphocytes in the Poly I:C and PMA groups showed a tendency to increase gradually until the end of the sampling period. The percentages of IgM^+^ B cells were highest on the 11th day after the injection of PMA, suggesting that IgM^+^ B cells have a major role in the Th2-related immune response in flounder. For the β-glucan group, the percentages of IgM^+^ B cells increased on the 1st day, reached a peak on the 7th day, and then gradually decreased. β-Glucan, as an immunostimulant, has been widely used in aquaculture for many years, and it can promote the production of IgM^37,40^. Therefore, the proliferation of IgM^+^ B cells was detected in the β-glucan group. However, IgM^+^ B cells gradually decreased on the 9^th^ day, and we suspect that some IgM^+^ B cells transformed into plasma cells, which do not express membrane-bound IgM on the cell surface^41^.

The differentiation fate of Th cells is governed predominantly by the cytokines in the microenvironment and the interaction of the T cell antigen receptor with antigen^42^. In addition, a corresponding positive feedback is formed during Th cell differentiation. For example, IFN-γ can promote Th1 cell differentiation, and IL-4 can promote Th2 cell differentiation. There is also mutual inhibition between Th subsets, which is achieved through interactions between key transcription factors. IL-12 and IFN-γ, which are required for Th1 cell differentiation, inhibit Th2 cell differentiation, whereas IL-4 inhibits Th1 cell differentiation^12,38^. In Atlantic cod, PMA increased the expression of GATA3 in vivo and in vitro, while there were no significant increases at the transcript level of GATA3 between the Poly I:C and β-glucan treatment groups^40^. The expression of rainbow trout T-bet and GATA3 in splenocytes was suppressed after stimulation with Poly I:C but upregulated after PMA treatment^43^. We found that Poly I:C could indeed upregulate the expression of T-bet, the major transcription factor of Th1 cells, as well as several Th1 cytokines, such as IFN-γ, IL-2 and IL-12. However, the transcription factors and cytokines corresponding to Th2 and Th17 types in CD4^+^ cells were suppressed. Similarly, PMA can upregulate GATA3 and IL-10, and β-glucan could upregulate ROR and IL-17 in CD4^+^ T cells. Notably, β-glucan also upregulated IL-2 and TNF-α, and TNF-a expression also increased after PMA stimulation. These results showed that flounder CD4^+^ cells have the potential to differentiate into different Th cells similar to mammals and are involved in Th-type immune responses. Further investigation is needed to explore the complex mechanism of CD4^+^ T cell differentiation in fish.

## Materials and methods

### Animals

Healthy flounders (*P. olivaceus*) of 20–25 cm were obtained from a fish farm in Rizhao, Shandong Province, China. The flounders were acclimated in recirculating seawater at 21 °C for a week and used for the following studies. Four- to six-week-old BALB/c mice were purchased from Qingdao Animal Experimental Center (Shandong, China), and they were used for monoclonal antibody (mAb) production.

### Selection and synthesis of epitope peptides

The protein sequences of flounder (*P. olivaceus*) CD4-1 and CD4-2 (accession No. BAM72051.1 and No. BAK40271.1) were retrieved from the NCBI database. The signal peptide, transmembrane region and immunoglobulin-like domains of CD4-1 and CD4-2 were predicted using SignalP (https://www.cbs.dtu.dk/services), TMHMM (https://www.cbs.dtu.dk/services) and SMART software (https://smart.embl-heidelberg.de/), respectively. The DNAStar Protean program (DNAStar, USA) was employed to predict the secondary structures and the physicochemical characteristics of amino acids, such as hydrophilicity, flexibility, surface accessibility, and antigenicity^44,45^. The potential epitopes of CD4-1 and CD4-2 were finally predicted by the online software Immune Epitope Database (IEDB; https://tools.immuneepitope.org/bcell/)^46^. Moreover, three-dimensional models of flounder CD4-1 and CD4-2 were created using the template model of the human T-cell surface glycoprotein CD4 in the Swiss-Model Repository Server (https://swissmodel.expasy.org/)^47^. Based on sequence analysis, the signal peptide and the sequences in the transmembrane and intracellular regions of flounder CD4-1 and CD4-2 were excluded from the immunogenic peptide candidates, and the peptide sequences in β-turns and random coil regions with good hydrophilicity, high accessibility, high flexibility and strong antigenicity were considered potential candidates. In addition, the three-dimensional models of flounder CD4-1 and CD4-2 were built to ensure that the peptide candidates were completely exposed on the surface of the structure model. Finally, the selected flounder CD4-1 and CD4-2 peptides were artificially synthesized by GenScript Biotechnology (Nanjing, China) with a purity of ≥ 90% and conjugated with keyhole limpet hemocyanin (KLH). The conjugated peptide was employed as an immunogen to produce mAbs against flounder CD4-1 and CD4-2 molecules.

### Production of monoclonal antibodies

The mAbs against flounder CD4-1 and CD4-2 peptides were produced as previously described^48–51^. In brief, two BALB/c mice were each immunized by intraperitoneal injection with 100 µl of 1 mg/ml KLH-conjugated peptide emulsified with an equivalent volume of Freund's complete adjuvant (Sigma, St. Louis, MO, USA). Two weeks later, a similar injection was administered using Freund's incomplete adjuvant instead of Freund's complete adjuvant. Then, booster injections with 100 µg KLH-conjugated peptide in 100 µl PBS were given twice by tail vein injection at 1-week intervals. Three days after the last injection, the spleen cells from the immunized mouse were fused with myeloma cells (P3-X63-Ag8U1). After culturing for 12–14 days in GIT medium (Nihon Seiyaku Co., Japan) supplemented with 1% HAT (Gibco, USA), the resultant hybridomas were first screened by antigen-specific ELISA as previously described in our lab^52^. Briefly, the wells of flat-bottom microplates (96-wells, Costar, USA) were coated with CD4-1 and CD4-2 free peptide, and the hybridoma culture supernatants were incubated as the primary antibody. Myeloma culture supernatant instead of primary antibodies was used as a negative control. The hybridomas that were positive in peptide ELISA were cloned by the method of limiting dilution repeated three times.

### Specificity analysis of mAbs

The target specificity of mAbs was confirmed by western blotting, mass spectrometry analysis, immunofluorescence staining and flow cytometry. The leukocytes were isolated from peripheral blood, spleen and head kidney of healthy flounder according to the method described previously^30^. The mAbs against flounder IgM were also used to localize the lymphocyte population size and granularity on the forward scatter (FSC)/side scatter (SSC) plots in flow cytometry analysis. The purified flounder CD3, CD4-1, CD4-2 and CD8β recombinant proteins were previously prepared in our lab^18,52^. The peripheral blood leukocyte lysates and recombinant proteins were subjected to SDS-PAGE and transferred onto PVDF membranes (Merck Millipore, Darmstadt, Germany). Then, the membranes were blocked and incubated with the anti-CD4-1 or anti-CD4-2 mAb as a primary antibody (diluted 1:1,000 in PBS). Antibody binding was detected with goat-anti-mouse Ig-alkaline phosphatase conjugate (diluted 1:4,000 in PBS) (Merck Millipore, Darmstadt, Germany). Finally, the bands were detected with freshly prepared substrate solution in the dark and stopped by washing with distilled water. The immunoreactive proteins in leukocyte lysates were excised from corresponding polyacrylamide gels and analyzed by mass spectrometry. The mass spectrometry analysis experiments were performed by Sangon Biotech (Shanghai, China). Indirect immunofluorescence staining and flow cytometry were also performed as previously described^53^, in which the rabbit anti-CD3ɛ polyclonal antibody^54^ (diluted 1:1,000 in PBS) was mixed with mouse anti-CD4-1 or CD4-2 monoclonal antibody (diluted 1:1,000 in PBS) as the primary antibody. The indirect immunofluorescence staining results were observed under a fluorescence microscope (Olympus DP70, Tokyo, Japan), and the results of flow cytometry were analyzed on an Accuri C6 flow cytometer (BD, USA). The mixture of unimmunized rabbit serum and myeloma culture supernatant was used as a negative control.

### Isolation of CD4^+^ T lymphocytes

In our previous study, CD4-1^+^/CD4-2^+^ (CD4DP) cells were found to be a major CD4^+^ T cell subpopulation in flounder^18^. Therefore, to obtain all CD4^+^ cells in peripheral blood leukocytes, the leukocytes were incubated with a mixture of CD4-1 and CD4-2 mAbs as the primary antibody. The method of isolation of CD4^+^ T cells was performed as previously described^55^. Briefly, after the primary antibody, the leukocytes were incubated with goat anti-mouse IgG magnetic beads (Miltenyi Biotech, Germany). Then, the leukocyte pellets were suspended in MACS buffer and flowed through a MACS LS column, and the magnetically unlabeled leukocytes were washed off using MACS buffer. The magnetically labeled leukocytes were collected, and to obtain high-purity CD4^+^ T lymphocytes, the magnetically labeled leukocytes were secondarily sorted using new equilibrated MACS LS columns. To analyze the purity of sorted cells, the cells were stained with Alexa Fluor 647-conjugated goat anti-mouse IgG antibody and analyzed by flow cytometry and indirect immunofluorescence assay. Moreover, the total RNA of sorted CD4^+^ cells was extracted, and expression analysis of cell marker genes was conducted by real-time PCR (RT-PCR)^56^.

### Poly I:C, PMA and β-glucan stimulation in vivo

Fish were randomly divided into four groups, and three groups of fish were injected intraperitoneally with 50 μl of 1 mg/ml Poly I:C, PMA or β-glucan. Fish in the control group were injected with 50 μl PBS. Leukocytes from peripheral blood, spleen and head kidney of flounder were sampled from each group at days 1, 3, 5, 7, 9 and 11 after injection. Then, the leukocytes were incubated with mAbs against CD4-1, CD4-2, IgM^48^ or mouse polyclonal antibody against CD8β^18^. After the leukocytes were incubated with the secondary antibody, the variations in CD4-1^+^, CD4-2^+^, IgM^+^ and CD8^+^ cells were analyzed by flow cytometry. Moreover, CD4^+^ T cells were isolated from peripheral blood by using an immunomagnetic bead separation method at 0, 1 and 3 days, and then total RNA of CD4^+^ cells was extracted for quantitative real-time PCR (q-PCR) analysis. The experiments were repeated three times.

### Quantitative real-time PCR (q-PCR)

cDNA from CD4^+^ cells was used as a template, and the expression of master transcription factors of T-helper (Th) cells and the gene expression of several cytokines were quantified using q-PCR. q-PCR was carried out using SYBR Green I Master Mix (Roche, Switzerland) in a LightCycler 480 II Real-Time System (Roche, Switzerland) as previously described^19^. Each sample was assessed in triplicate with the 18S gene as an internal control. All data were analyzed with the 2^-ΔΔCt^ method.

### Statistics

The experiments in this study were repeated in triplicate, and the data are expressed as the mean ± SEM. *P* values < 0.05 were considered significant. One-way analysis of variance (ANOVA) and Duncan’s multiple comparisons were performed by using Statistical Product and Service Solution (SPSS) software (version 20.0; SPSS, IBM, BY, USA).

### Ethics statement

The experiments with animals used in the present study were carried out strictly in line with the procedures in the Guide of the Use of Experimental Animals of the Ocean University of China. More importantly, the methods used in the animal experiments of this study were approved by the Instructional Animal Care and Use Committee of the Ocean University of China (permit number: 20150101). All possible efforts were made to minimizing suffering.

## Supplementary information

Supplementary file1 (PDF 546 kb)

## Data Availability

The datasets generated during and/or analysed during the current study are available from the corresponding author on reasonable request.
