# Supplementary material for: Kinetics of T lymphocyte subsets and B lymphocytes in response to immunostimulants in flounder (Paralichthys olivaceus): implications for CD4+ T lymphocyte differentiation
Source: Sci Rep. 2020 Aug 14;10:13827. doi: 10.1038/s41598-020-69542-6 (PMC7429840; doi:10.1038/s41598-020-69542-6)

Kinetics of T lymphocyte subsets and B lymphocytes in response to immunostimulants in flounder (*Paralichthys olivaceus*): Implications for CD4$^+$ T lymphocyte differentiation

Jing Xing[1,2], Hong-fei Tian[1], Xiao-qian Tang[1], Xiu-zhen Sheng[1], Wen-bin Zhan[1,2,*]

1. Laboratory of Pathology and Immunology of Aquatic Animals, KLMME, Ocean University of China, Qingdao 266003, P. R. China

2. Laboratory for Marine Fisheries Science and Food Production Processes, Qingdao National Laboratory for Marine Science and Technology, No. 1 Wenhai Road, Aoshanwei Town, Qingdao, China

*Corresponding author: wbzhan@ouc.edu.cn

Tel: +86-532-82032284; fax: +86-532-82032284

Supplementary information:

Table S1.

Primers used in this study.

Primer name	Primer sequence (5′→3′)	Accession
RT-PCR		
TCRα-F	TCTCCAGGACAATAAAGAGTAGTGA	AB053227.1
TCRα-R	TGCACAGACCTCAGATTCATT	
TCRβ-F	CCCCACGACATCTCAAG	AB053228.1
TCRβ-R	GTCTGGACCTTGTTCACC	
CD3ε-F	ATGACCGGGACGATAATTCTGATGA	AB081751.1
CD3ε-R	CATAGTCAGGAGATGGGACAGGTGG	
CD4-1-F	CACCCTAAAGCCTCAAGTGGAAAT	AB643634
CD4-1-R	AAGTTTTCTGGTTGGATTTGTTTGA	
CD4-2-F	TTGGTGGGGTGATGTACACAGAG	AB640684
CD4-2R	CACAGTTGGGGCACGATGTCTC	
CD8α-F	GGTGAAACCAGTTCTATCATCCCTT	AB082957
CD8α-R	TGGTGGTGCGGGCATCTC	
CD8β-F	GTCACCCGAAGAAGAATTTTGC	AB643633

CD8β-R	ATCTTCTGAAAGTGGTGGCG	
IgM-F	GAACTGAAAGTGTCTGCCTTCTATG	AF226284
IgM-R	CCATTCTCGCTTTTATGTTCCTC	
IgD-F	TGGGGACAAGGGACAAAGG	AB052658
IgD-R	GCGAGGCAGCCAAGAGTG	
IgT-F	TAATTGTTCAGTAACTCATGCCG	KX174301
IgT-R	GATTGAAGTGTTCCTATGCGTCT	
CD79β-F	GCAGCATCAGAATAGCGACA	KC345763.1
CD79β-R	TGTTCCTCAAAGCCACCTCTGCC	
CD83-F	CCAACGGCACGACGACATACT	KR998304.1
CD83-R	GAGGGCAACCATCAGCACAAC	
CSF-1R-F	AAATGTAACCACCCTCACTTGC	XM_020082410.1
CSF-1R-R	TCTTCCTTTCCCAGACCGTAA	
β-actin-F	GATGGTGGGTATGGGCCAGAAG	HQ386788.1
β-actin-R	ATGTCACGCACGATTTCCCTCTC	
qPCR		
T-bet-F	GCCGACATCAGCAGTCACCT	KR822591.1
T-bet-R	TGTGCGTAAAACCTGCCG	
IFN-γ-F	TGTCAGGTCAGAGGATCACACAT	AB435093
IFN-γ-R	GCAGGAGGTTCTGGATGGTTT	
IL-2-F	ATGGAGCACTTTATTGGGATT	KY307833
IL-2-R	TCACATTTGTTGGAGCGTAGA	
IL-12α-F	ACTGTTCTGCTCAAATCCCTCAT	XM_020101876.1
IL-12α-R	GTTCATGTATCCGAAGACCCTGT	
IL-12β-F	TAAATCAATTCTCTAGATGGCGCAG	XM_020097141.1
IL-12β-R	CGGTCAAGTCCATTTGCTCCTAT	
GATA3-F	CAGGAGGACAAAGAGTGCATAAAGT	XM_020108979.1
GATA3-R	GAAGATGACCCACCTATCAGGCTAC	
IL-10-F	TACGAAGCGAACGATGACCTA	XM_020086558.1
IL-10-R	GCTCGTCGAAGATTTGCTGTAT	
RORα-F	GCACCTGGCCCAGAACATC	XM_020079419.1
RORα-R	TTGGCGAACTCCACCACATAC	
IL-17A-F	CCTGGATGTGACTCCTTGTTGG	XM_020111881.1
IL-17A-R	GACGCTCTGGTAGATGGGAACT	
IL-17C-F	GACGAGGCTGCGGAGAAGAAG	XM_020112925.1
IL-17C-R	GAGTAGCTGACGGGGAAGTGA	
IL-17D-F	GAGCCGCAGGACAAACTGAAC	XM_020100689.1
IL-17D-R	TCCCATCAGGCAGCCCTTA	
TNF-α-F	GTCCTGGCGTTTTCTTGGTA	AB040448
TNF-α-R	CTTGGCTCTGCTGCTGATTT	
18sRNA-F	GGTCTGTGATGCCCTTAGATGTC	EF126037

18sRNA-R	AGTGGGGTTCAGCGGGTTAC

Fig.S1

The original and unprocessed gel (a) and blots (b) figures are used in Fig.2.

(a)

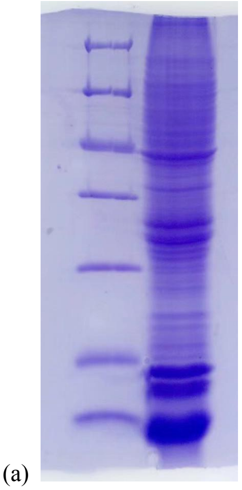

(b)

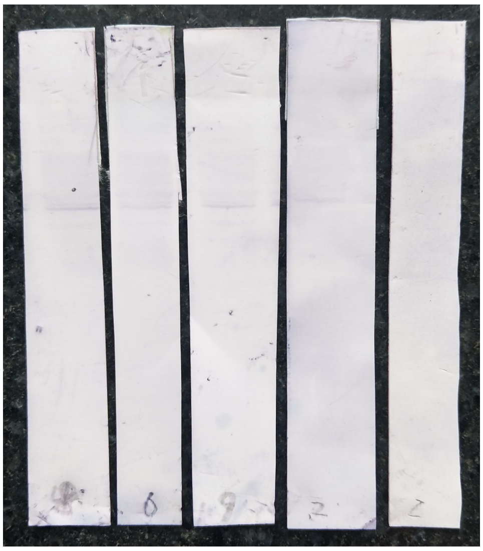

Fig.S2

The original and unprocessed gel (a) and blots (b) figures are used in Fig.3.

(a)

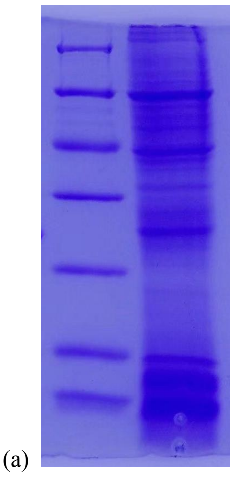

(b)

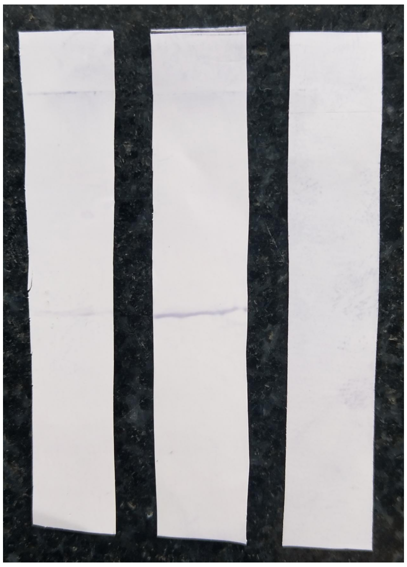

Fig.S3

The original and unprocessed gel figures are used in Fig.7.

CD4^{+} cells

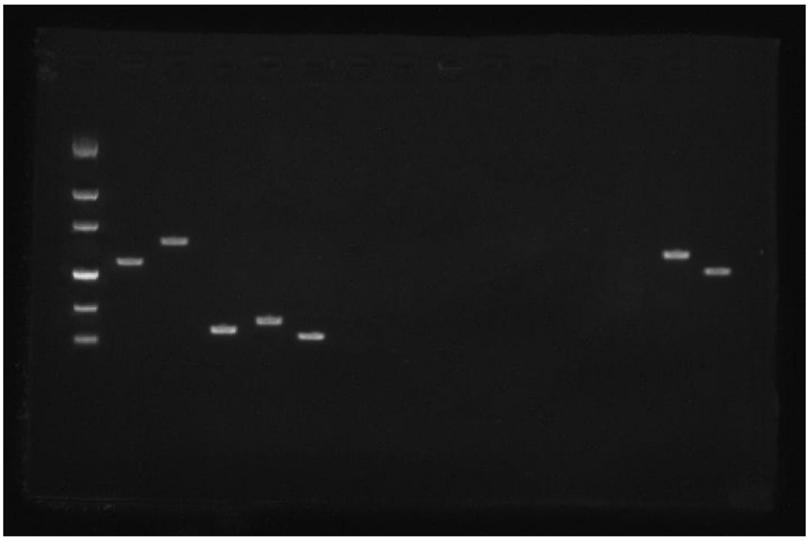

CD4^{-} cells

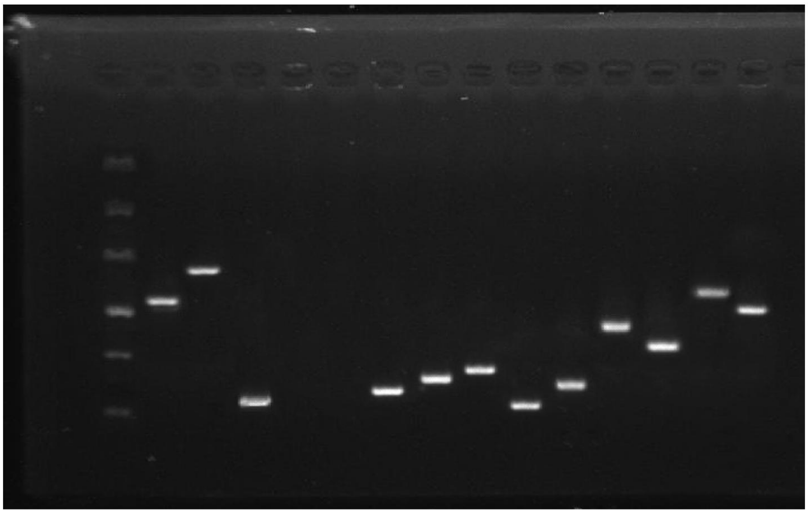

Supplement: Supplementary file 1 — Supplementary file1 (PDF 546 kb) [file 41598_2020_69542_MOESM1_ESM.pdf]
